# Predicting Affective Information – An Evaluation of Repetition Suppression Effects

**DOI:** 10.3389/fpsyg.2016.01365

**Published:** 2016-09-09

**Authors:** Sabrina Trapp, Sonja A. Kotz

**Affiliations:** ^1^Gonda Multidisciplinary Brain Research Center, Bar-Ilan University, Ramat GanIsrael; ^2^Max Planck Institute for Human Cognitive and Brain Sciences, LeipzigGermany; ^3^Faculty of Psychology and Neuroscience, Maastricht University, MaastrichtNetherlands

**Keywords:** predictive coding, bottom-up attention, habituation, anxiety disorders, emotion, social phobia

## Abstract

Both theoretical proposals and empirical studies suggest that the brain interprets sensory input based on expectations to mitigate computational burden. However, as social beings, much of sensory input is affectively loaded – e.g., the smile of a partner, the critical voice of a boss, or the welcoming gesture of a friend. Given that affective information is highly complex and often ambiguous, building up expectations of upcoming affective sensory input may greatly contribute to its rapid and efficient processing. This review points to the role of affective information in the context of the ‘predictive brain’. It particularly focuses on repetition suppression (RS) effects that have recently been linked to prediction processes. The findings are interpreted as evidence for more pronounced prediction processes with affective material. Importantly, it is argued that bottom-up attention inflates the neural RS effect, and because affective stimuli tend to attract more bottom-up attention, it thereby particularly overshadows the magnitude of RS effects for this information. Finally, anxiety disorders, such as social phobia, are briefly discussed as manifestations of modulations in affective prediction.

## Predictive Brain

A major purpose of science is the formulation of fundamental laws. Recently, a framework with similar scope has been suggested in the field of neuroscience. The key idea is that surprise minimization is a major computational goal of the brain, and that this gives rise to functions like perception, action, attention and memory (Friston, 2005, 2010). In this framework, surprise minimization equals minimization of prediction error, and consequently, it has been suggested that the brain constantly predicts its own sensory input. Decreased neural responses for predictable sensory events are considered as the hallmark of predictive processing, and indeed they have been reported in numerous studies (e.g., Alink et al., 2010; Todorovic et al., 2011; de Gardelle et al., 2013). Predictions about sensory events can be generated by cues, temporal and spatial regularities, and via semantic, contextual, and associative processes (Bar, 2007; Turk-Browne et al., 2010; Kimura et al., 2012; Kok et al., 2014; Bendixen et al., 2015; Jessen and Kotz, 2015; Trapp et al., 2016). But expectations can also emerge as a consequence of repetitive exposure. A reduced neural signal when a stimulus is repeated is not an altogether new finding. This phenomenon was termed *repetition suppression* (RS; for a review see Grill-Spector et al., 2006). Several models have been proposed that attempt to explain this effect, e.g., neural fatigue and adaptation (for a discussion see Larsson and Smith, 2012) or sharpening of representation (Freedman et al., 2006). More recently, the RS effect has also been linked to predictive processing. Summerfield et al. (2008) showed that RS is attenuated when repetitions become less predictable, and suggested that the effect may be linked to the process of prediction error minimization. Hereafter, several modulating factors of this interaction between RS and predictive processing have been identified (for a review see Grotheer and Kovács, 2016).

## Special Status of Affective Information

Often, the stimuli used in RS (or other paradigms that tap into prediction processes) were neutral. However, as social beings, much of the sensory input in our daily life is affectively loaded. The importance of affective information in human social interactions and communication strongly suggests that this information must be processed extremely fast and efficiently. Especially in the context of human communication, affective facial and vocal expressions as well as body gestures may serve as an additional source of information to support comprehension and to avoid misinterpretations, e.g., predicting that the subtle twitching of the corner of a mouth will develop into a smile can help to interpret the upcoming verbal expressions as ironic.

There is good reason to assume that prediction processes differ between affective and neutral information. Biologically or socially relevant information (e.g., spiders, fearful faces) is particularly salient, is detected faster (Ohman et al., 2001) and harder to ignore even when contextually irrelevant (Richards and Blanchette, 2004). A neural correlate of these behavioral findings is that processing of affective stimuli consistently elicits stronger activation in occipito-temporal areas (Vuilleumier et al., 2001; Pessoa et al., 2002; Sabatinelli et al., 2005). There are similar reports for vocal information (Schirmer and Kotz, 2006; Brück et al., 2011). Furthermore, it has been shown that affective stimuli modulate both early sensory and later components in event-related potential studies (Kotz and Paulmann, 2007; Pourtois et al., 2010).

Furthermore, there is evidence for neural networks that specifically subserve the prediction of affective stimulus material. Bermpohl et al. (2006) used stimuli depicting various neutral or emotional scenes. A cue announced whether the upcoming picture is neutral or affective; for comparison, the picture was presented without any preceding information. The authors identified brain regions that are specifically linked to affective predictions, and that are not associated with emotion perception or arousal. Feldman-Barrett and Bar (2009) suggested that the brain routinely makes affective predictions in visual recognition. The authors defined ‘affective information’ as input that influences a person’s vascular body reactions, such as heart rate and hormonal secretions. They posited that during object recognition, the current input is not only compared with already stored perceptual events, but this process also considers prior affective experience, i.e., how the perceptual input has influenced bodily sensations. They argued that the idea is supported by anatomical connections of the medial orbitofrontal cortex, which plays a role in expectancy-based processes and has projections to the hypothalamus, midbrain, and brainstem, and may thereby modify the perceiver’s body state.

## Repetition Suppression and Affective Information

Here, we intend to exemplify differences between neutral and affective prediction with the RS paradigm. In the context of predictive coding, the reduced neural signal for the repeated stimulus is interpreted as a manifestation of prediction error minimization. In the predictive coding framework, all sensory input needs to be explained, and what cannot be explained, will be treated as prediction error. The repeated stimulus was predicted, and therefore, the prediction error decreases. In this framework, a *stronger* RS effect translates to a lower prediction error, and more or more accurate predictions. A *weaker* RS effect translates to a higher prediction error, and fewer or less accurate predictions.

Importantly, there are differences in RS effects between neutral and affective stimuli. Gerlicher et al. (2014) used electroencephalography and evoked steady-state visual potentials and found that adaptation effects *decreased* linearly with negative valence. Using fMRI, Rotshtein et al. (2001) reported that the repetition of unpleasant faces was associated with *less* RS in occipital-temporal cortex. Similarly, Suzuki et al. (2011) found that neutral faces elicited larger RS in ventral visual cortex, fusiform gyrus, and right inferior occipital gyrus, while the effect was *absent* or *attenuated* for happy faces. The authors explained these effects with sustained neural processing of happy faces, possibly at the stage of encoding and identification.

In the context of predictive processing, less RS would translate to less prediction error minimization, indicating fewer predictions, or predictions that are less accurate. However, fewer or less accurate predictions for affective material seem counter-intuitive: Predictions allow the preparation of responses and rapid reactions; therefore, one would expect more or more accurate predictions for affective stimuli. Indeed, there is also evidence for a *larger* RS effect with affective material. Ishai et al. (2004) have demonstrated that fearful faces lead to larger RS effects as compared to neutral faces. Similarly, Ethofer et al. (2009) used words that were either angry or neutral and found larger RS effects for affective stimuli in the OFC.

## Task Difficulty As a Modulating Factor in Repetition Suppression

How can these contradictory findings on RS and affective information be reconciled? Why do some studies report more and other studies less RS with affective stimuli? It is important to note that the neural correlates of RS may be confounded by other processes that are not linked to prediction and/or adaptation effects. For example, it is well known that bottom-up attention increases the reduced blood-oxygen level dependant (BOLD) response in fMRI (McMains and Kastner, 2011). However, an increase of the BOLD response would *reduce* the magnitude of the RS effect. As affective stimuli tend to attract more bottom-up attention (for a review see Carretié, 2014), the BOLD response may be inflated, and consequently, the effect of prediction error minimization may be overshadowed.

A possible factor that may affect the amount of bottom-up attention could be the task difficulty. Merely passive viewing, such as in the study by Suzuki et al. (2011), may generally enable more influences from bottom-up. A more difficult task, in contrast, may bias the brain toward expected and relevant aspects in the environment, and thereby decrease influences from bottom-up. For example, if the task is a variant of a short-term memory paradigm, like in the study by Ishai et al. (2004), the brain may generally increase expectations of affective facial expressions, so that those are not experienced as disturbing, and the decision can be done fast and accurately. Such a default high expectation increases the RS effect.

It will be important to look at differences between negative and positive stimuli. It has been reported that a positive affect broadens the scope of attention, and fosters more global information processing (Basso et al., 1996; Gasper and Clore, 2002; Fredrickson and Branigan, 2005; Rowe et al., 2007). Therefore, the prediction of positive input may enable a broader, but possibly less precise window into the future. Conversely, negative mood has been linked to a more constricted focus of attention that lacks peripheral details (Gable and Harmon-Jones, 2010). This may promote more precise predictions, which may, however, be more limited in scope.

In sum, in line with recent suggestions for neutral material (Auksztulewicz and Friston, 2016), we suggest to re-interpret the RS effects with affective stimuli within the framework of predictive processing. Intuitively, one would expect better predictions for affective material, and this is supported by larger RS effects. However, also the opposite finding was reported – a decreased RS for affective information. We here draw attention to a possible confound because affective material may attract more bottom-up attention, and this can *overshadow* the magnitude (and possibly the direction) of the RS effect. We propose that it is only when the task is very easy, and influences from bottom-up increase, that affective stimuli show *less* RS than neutral stimuli. The presence of potential modulating factors does not negate potential contributions of predictive processing, but their existence should be considered with great caution in the interpretation of RS effects. Prediction error minimization and bottom-up attention act as opposing factors on the neural RS effect. Future studies may directly test this idea by varying task difficulty in the context of RS paradigms and affective stimulus material.

As of yet, there are only a few studies on RS and affective material, and it will be crucial to gather more evidence to either support or falsify our proposal here. It should also be noted that there are other paradigms that tap into prediction processes, such as using cues, manipulating temporal or spatial regularities or predictions that are elicited by associations (Bar, 2007; Turk-Browne et al., 2010; Kimura et al., 2012; Kok et al., 2014). Furthermore, there is evidence for dissociable expectation suppression and RS effects in the auditory and visual cortex both for the location of effects and for their temporal onset (Todorovic and de Lange, 2012; Grotheer and Kovács, 2015). It will therefore be important to examine to what extend differences in expectation suppression between affective and neutral stimuli are comparable with RS.

## Clinical Implications

Elucidating the mechanism of affective prediction is also important for our understanding of psychiatric and psychological disorders. Recently, it has been suggested that excessive anticipatory responding is a common feature among several anxiety disorders (Grupe and Nitschke, 2013). Modulations in affective prediction may be a driving force in the etiology and persistence of anxiety disorders. For example, Sladky et al. (2012) investigated performance in a facial discrimination task with participants suffering from social anxiety disorder (SAD). They found suppression effects for affective stimuli in patients with SAD in the amygdala, OFC, pulvinar, and thalamus, while no such suppression was found in healthy controls. If suppression effects are partly due to predictive processes, this finding can be explained by SAD participants making more precise affective predictions, presumably to achieve higher control, and to be less (negatively) surprised. This idea is also supported by the finding that when threatened by the probability of an electric shock in a block of trials, participants with higher scores in trait anxiety show improved perceptual sensitivity if they anticipated fearful stimuli (Sussman et al., 2016). Furthermore, it is conceivable that SAD individuals need less information to predict an affective (particularly negative) trajectory, e.g., that of an evolving negative facial expression. This aligns with the finding that for individuals suffering from social phobia, a less intense facial expression is sufficient to correctly identify anger in comparison to depressive and healthy participants (Joormann and Gotlib, 2006). These individuals may well be able to better extrapolate this expression into the future. However, as outlined above, negative predictions may be generally more focused. A default negative expectation could therefore ignore or suppress predictions about upcoming positive events, and thereby bias perception toward negative events, which contributes to the persisting experience of threat. RS paradigms allow probing for differences in affective prediction, and may thereby not only elucidate our understanding of the disorder, but also serve as a precious diagnostic tool in clinical contexts.

## Summary

As social beings, we are constantly exposed to affective information. Such input is often ambiguous. Expectations facilitate dealing with noisy and ambiguous input, and foster rapid and efficient interpretation. As of yet, the role of affective information has received little attention in models of predictive processing. While the divergence of findings regarding affective RS warrants further investigation, data point to the importance of affective information in the context of predictive processing. Future studies will have to gather more data on RS and affective information as well as determine the role of affective information in other prediction paradigms to elucidate the underlying mechanisms. Finally, with more data, RS may serve as a clinical tool to assess differences and deficits in affective predictions.

## Author Contributions

ST conceived and wrote the paper, and SK provided revisions.

## Conflict of Interest Statement

The authors declare that the research was conducted in the absence of any commercial or financial relationships that could be construed as a potential conflict of interest.
